# Developing and validating a clinical algorithm for the diagnosis of podoconiosis

**DOI:** 10.1093/trstmh/traa074

**Published:** 2020-11-11

**Authors:** Kebede Deribe, Lyndsey Florence, Abebe Kelemework, Tigist Getaneh, Girmay Tsegay, Jorge Cano, Emanuele Giorgi, Melanie J Newport, Gail Davey

**Affiliations:** Brighton and Sussex Centre for Global Health Research, Department of Global Health and Infection, Brighton and Sussex Medical School, Brighton, BN1 9PX, UK; School of Public Health, College of Health Sciences, Addis Ababa University, Addis Ababa, PO Box 9086, Ethiopia; King's College Hospital NHS Foundation Trust, Denmark Hill SE5 9RS, London, UK; International Orthodox Christian Charities, PO Box 495 Bahir Dar, Ethiopia; International Orthodox Christian Charities, PO Box 495 Bahir Dar, Ethiopia; College of Medicine and Health Sciences, Debre Markos University, PO Box 269, Debre Markos, Ethiopia; Department of Disease Control, London School of Hygiene & Tropical Medicine, WC1E 7HT, London, UK; CHICAS Research Group, Lancaster Medical School, Lancaster University, Bailrigg, LA1 4YW, Lancaster, UK; Brighton and Sussex Centre for Global Health Research, Department of Global Health and Infection, Brighton and Sussex Medical School, Brighton, BN1 9PX, UK; Brighton and Sussex Centre for Global Health Research, Department of Global Health and Infection, Brighton and Sussex Medical School, Brighton, BN1 9PX, UK; School of Public Health, College of Health Sciences, Addis Ababa University, Addis Ababa, PO Box 9086, Ethiopia

**Keywords:** diagnosis, clinical algorithm, clinical decision algorithms, Ethiopia, lymphoedema, podoconiosis

## Abstract

**Background:**

Difficulties in reliably diagnosing podoconiosis have severely limited the scale-up and uptake of the World Health Organization–recommended morbidity management and disability prevention interventions for affected people. We aimed to identify a set of clinical features that, combined into an algorithm, allow for diagnosis of podoconiosis.

**Methods:**

We identified 372 people with lymphoedema and administered a structured questionnaire on signs and symptoms associated with podoconiosis and other potential causes of lymphoedema in northern Ethiopia. All individuals were tested for *Wuchereria bancrofti*–specific immunoglobulin G4 in the field using Wb123.

**Results:**

Based on expert diagnosis, 344 (92.5%) of the 372 participants had podoconiosis. The rest had lymphoedema due to other aetiologies. The best-performing set of symptoms and signs was the presence of moss on the lower legs and a family history of leg swelling, plus the absence of current or previous leprosy, plus the absence of swelling in the groin, plus the absence of chronic illness (such as diabetes mellitus or heart or kidney diseases). The overall sensitivity of the algorithm was 91% (95% confidence interval [CI] 87.6 to 94.4) and specificity was 95% (95% CI 85.45 to 100).

**Conclusions:**

We developed a clinical algorithm of clinical history and physical examination that could be used in areas suspected or endemic for podoconiosis. Use of this algorithm should enable earlier identification of podoconiosis cases and scale-up of interventions.

## Introduction

Podoconiosis is a chronic inflammatory disease caused by long-term contact with irritant minerals in the soil.^1^ The disease causes swelling of the lower leg, which interferes with the day-to-day activities of affected individuals.^2^ The major signs and symptoms of the disease include lymphoedema of the lower legs and acute dermatolymphangioadenitis (ADLA), which is characterised by malaise, fever, chills, diffuse inflammation, swelling of the limbs, lymphangitis, adenitis and eventually skin peeling.^3^ The disease has major social and economic consequences through stigma and loss of productivity.^4,5^ The disease can be prevented by consistent use of footwear, foot hygiene and use of floor coverings.^2^ The disease can be treated in its early stages by using simple management of the lymphoedema-related morbidity, which includes foot hygiene, skin care, wound care, compression, exercises, elevation of the legs and treatment of acute infections.^3,6^ Despite the formidable challenges the disease is presenting in endemic countries, there is no proven point-of-care diagnostic method for the disease.^7,8^ The diagnosis of the disease largely depends on clinical diagnosis and exclusion of other potential causes of lymphoedema.^9,10^

Although clinical diagnosis of the disease has been used for several years,^11^ there is no validated clinical algorithm for diagnosis of the disease.^9^,^11^ The diagnostic approach used to map podoconiosis in Ethiopia, Cameroon and Rwanda was based on excluding other potential causes of lymphoedema. The survey conducted in Ethiopia used history and physical examination to exclude other causes of lymphoedema, including systemic disease, leprosy, onchocerciasis and hereditary causes of lymphoedema, and antigen-based immunochromatographic card tests (ICTs) to exclude lymphatic filariasis (LF).^9,12^ In Cameroon, clinical history, physical examination and certain disease-specific tests described above were used to exclude common differential diagnoses to reach the diagnosis of podoconiosis.^13^ All individuals with lymphoedema included in the survey were tested for circulating *Wuchereria bancrofti* antigen and specific immunoglobulin G4 (IgG4) in the field using, respectively, the Alere Filariasis Test Strip (FTS) test^14^ and the SD BIOLINE LF IgG4 test (Wb123),^15^ in addition to night thick blood films. DNA specific to *W. bancrofti* was tested on night blood using the quantitative polymerase chain reaction technique.^13^

Even though clinical diagnosis based on signs and symptoms is frequently used, the way in which each of the clinical features of podoconiosis predicts true diagnosis and the minimum requirements of signs and symptoms needed to reach the diagnosis of podoconiosis have not been statistically validated.^7^ The accurate identification of lymphoedema due to podoconiosis in areas where other causes of lymphoedema, such as LF, are common remains a challenge for primary healthcare workers.^9^ Although there are point-of-care diagnostic tests for LF, which can easily be implemented during large-scale surveys, these tests are costly for routine practice and have low sensitivity in advanced cases.^9^

Early intervention among podoconiosis patients is important in preventing the progression of lymphoedema.^6^ For early intervention to be optimally effective, early detection is vital.^7,16^ As healthcare providers become progressively more involved in examination, evaluation and early intervention programs, increasing the accuracy of these examinations will be essential to optimize these interventions. Evidence has shown that morbidity management reduces disease severity and the frequency of ADLA episodes, resulting in fewer days off work and an overall improvement in quality of life.^3,17^ In addition, diagnosing podoconiosis in an individual provides an opportunity for counselling family members (there is evidence of a genetic influence in susceptibility to the condition)^18^ and instigating primary prevention strategies. Our study aimed to develop a diagnostic algorithm based on clinical features easily identifiable by primary healthcare workers.

## Materials and methods

### Study setting

**Figure 1.**
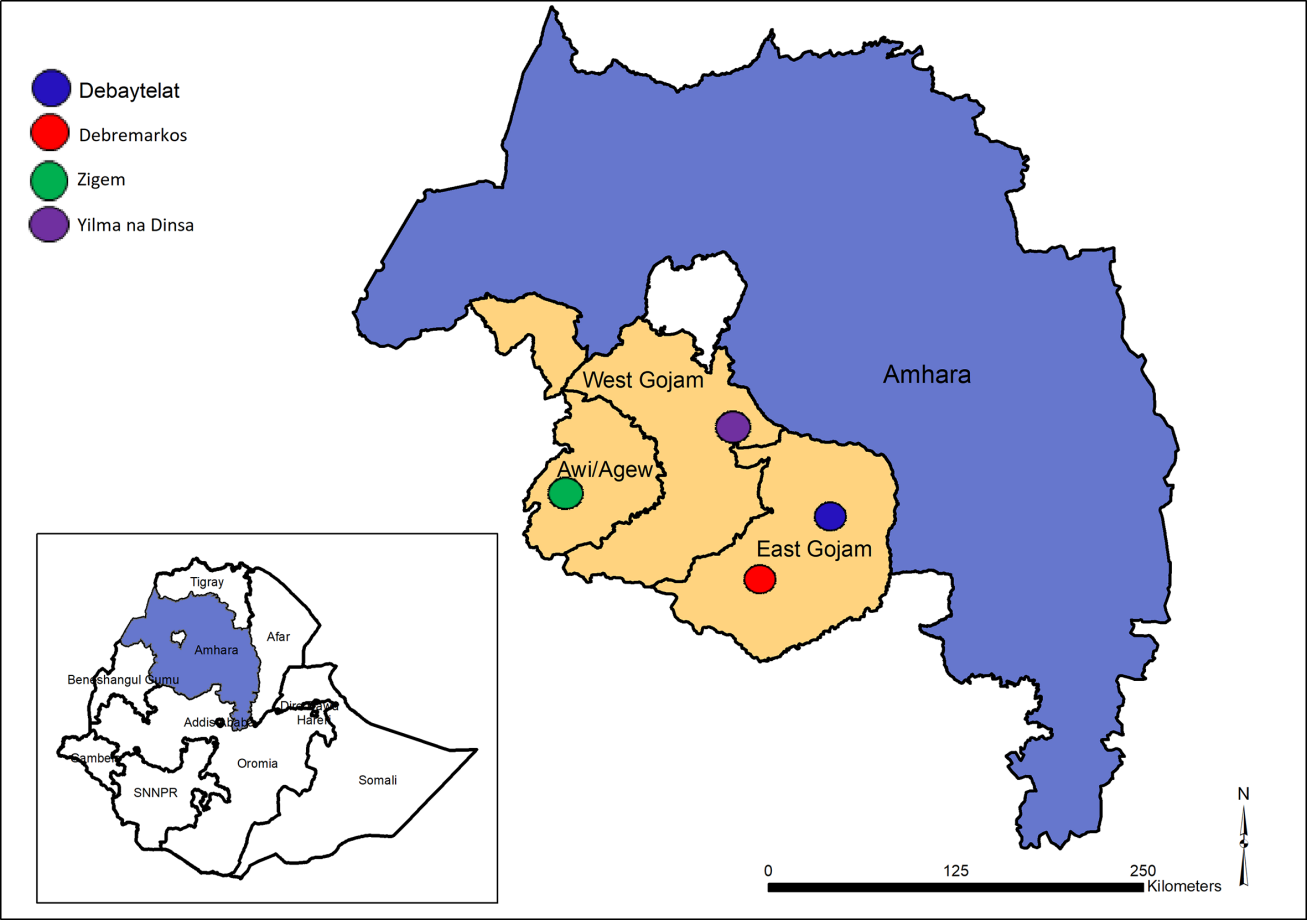
Map of the study locations.

The study was conducted in Ethiopia, within four *woreda* (districts) in the Amhara region of northern Ethiopia (Figure 1) in March 2017. These were selected to include areas of both high and low prevalence of podoconiosis and areas where podoconiosis and LF are co-endemic. Zigem is an LF-endemic district in Awi zone,^19,20^ in which mass drug administration using albendazole and ivermectin was started in 2015. The district is also of low endemicity for podoconiosis (1–5%).^21,22^ Yilma na Dinsa is highly endemic for podoconiosis (>10%),^20^,^21^ but not for LF.^19,20^ Debaytelat and Debremarkos are endemic for podoconiosis, with low prevalence (1–5%),^21,22^ and are non-endemic for LF.^19,20^

### Study population

Participants were identified using convenience sampling. For each of the four *woreda* visited, individuals with leg swelling were mobilised to a local health centre in advance. Participants were deemed eligible if they had lymphoedema of the lower leg(s). Those who did not have lymphoedema of either of their lower legs and individuals <15 y of age were excluded from the study. The purpose and method of the study were explained in the local language.

### Data collection

For each participant, the diagnosis was established and documented by healthcare workers, all of them holding a bachelor's degree in nursing or health sciences and with ample experience in the diagnosis of podoconiosis and lymphoedema. Their final diagnosis was used as the reference standard.^11,13^ The healthcare workers used history, physical examination and Wb123 to exclude other causes and arrive at the diagnosis of podoconiosis. The data collectors were provided with a half-day orientation on the procedure of data collection and the tools to be used. Prospective data were collected using a standardised questionnaire that recorded demographic information, history and examination findings and Wb123 test results. The variables included in the index test questionnaire were selected on the basis that they could easily be assessed by primary healthcare workers and were relevant in establishing a diagnosis of podoconiosis. The Wb123 test was performed using the SD BIOLINE LF IgG4 test kit (Standard Diagnostic, Suwon, Korea) according to the manufacturer's instructions and results were read and recorded at both 30 min and 24 h.^13^ Briefly, 10 μL of capillary blood was obtained from each person by finger prick and applied to the sample application pad of the Wb123. A single test was performed for each participant by a trained laboratory technician. Before utilization of the test, positive and negative quality control tests were conducted as per the manufacturer's instructions.^15^

### Data analysis and algorithm construction

Descriptive analysis using a frequency table was conducted to present the findings. Univariate logistic regression was done to estimate the odds ratio (OR) to show potential association of the signs and symptoms with podoconiosis. To develop a predictive algorithm for diagnosis of podoconiosis, we developed a logistic regression model for the probability that the *i*th individual has a positive diagnosis, which we denote as *p_i_* for *i*=1, …, 372. Based on the logistic regression model, our model for *p_i_*, which includes all the explanatory variables, can be expressed as

(1) \begin{equation*}{\rm{log}}\ \left\{ {\frac{{{p_i}}}{{1 - {p_i}}}} \right\} = \sum\limits_{j = 1}^{14} {\,\,{\beta _j}{x_j}} ,\end{equation*}

where *x_j_* denotes the *j*th explanatory variable of the 14 considered in this study.

Let ${\hat{\beta }_j}\ $denote the maximum likelihood estimator for ${\beta _j}$ and ${\hat{p}_i}\ $that for ${p_i}$. Using the model in equation 1, we then classify an individual as positive if the following statement holds: ‘the probability, p, that ${\hat{p}_i}\ $exceeds a threshold R is greater than Q’, or more formally ${\rm{p}}\ = \ Prob[ {\ {{\hat{p}}_i} > R} ] > Q$. Note that this is mathematically equivalent to the statement ${\rm{p}}\ = \ Prob[ {\mathop \sum \nolimits_{j\ = \ 1}^{14} {{\hat{\beta }}_j}{x_j} > {\rm{log}}\{ {R/( {1 - R} )} \}} ] > Q$, hence to compute p we can use the multivariate Gaussian distribution of the maximum likelihood estimator for the ${\hat{\beta }_j}$.

In order to identify the best subset of covariates *x_j_* that yields the highest sensitivity and specificity, we proceed as follows. We consider all possible combinations of covariates, totalling 2^14^=16 384 logistic regression models, and for each of these we use leave-one-out cross-validation to generate the probability S for the *i*th individual excluded from the training set. Based on S, the model then classifies each of the 372 individuals while optimizing the parameters R and Q in order to maximize both the sensitivity and specificity of the model.

All the computations were carried out using the R software environment (version 3.6.3; R Foundation for Statistical Computing, Vienna, Austria).^23^

### Ethical considerations

Written informed consent was obtained for all participants. If a selected individual was between 16 and 18 y of age, verbal assent and permission were obtained from the study participant and a legal guardian, respectively. This included consent to undergo Wb123 testing. For those who could not write, informed consent was provided through a fingerprint.

All enrolled participants were given an education session regarding the diagnosis and management of podoconiosis, including a demonstration of how to wash their feet and legs regularly with soap and water.

## Results

A total of 372 participants with lymphoedema of the lower leg were included in the study. Most of them (252 [67.7%]) were from Yilma na Dinsa, 96 (25.8%) were from Zigem, 15 (4.1%) were from Debremarkos and the rest (9 [2.4%]) were from Debaytelat.

Fifty-one percent were male, 80% were from urban areas, 32% were able to read and write and 11% had attended formal education (Table 1).

**Table 1.** Sociodemographic characteristics of the participants

Characteristics	n (%)
Sex	
Male	191 (51.3)
Female	181 (48.7)
Age (years)	
16–24	17 (4.6)
25–34	45 (12.1)
35–44	71 (19.1)
45–54	105 (28.2)
55–64	85 (22.8)
≥65	49 (13.2)
Zone	
East Gojam	24 (6.5)
West Gojam	296 (79.6)
Awi	52 (14)
*Woreda*	
Zigem	96 (25.8)
Yilma na Dinsa	252 (67.7)
Debremarkos	15 (4.1)
Debaytelat	9 (2.4)
Residence	
Rural	74 (20)
Urban	296 (80)
Read and write	
Yes	119 (32.1)
No	252 (67.9)
Marital status	
Never married	25 (6.7)
Married	234 (63.1)
Divorced	83 (22.4)
Widowed	29 (7.8)

Table 2 shows the signs and symptoms recorded among people with lymphoedema. The majority of the cases had bilateral (321 [86.3%]), symmetrical (217 [67.8%]) and below-the-knee (362 [98.9%]) swelling, which started from the lower leg (362 [97.6%]), and slightly more than half (202 [54.3%]) had mossy changes. Very few people with hydrocele were identified (4 [2.1%]). For all 372 participants, the results of the Wb123 test were negative at both the 30-min and 24-h readings. Based on expert diagnosis, 344 of 372 participants (92.5%) were podoconiosis cases and the remaining 28 (7.5%) were due to lymphoedema of other causes.

**Table 2.** Clinical signs and symptoms among people with lymphedema

Variable	Categories	n (%)
Type of swelling	Unilateral	51 (13.7)
	Bilateral	321 (86.3)
Does the swelling differ in size between the two legs?	Yes	103 (32.2)
	No	217 (67.8)
Is the swelling limited to below the knee?	Yes	362 (98.9)
	No	4 (1.1)
Where did the swelling start from?	Lower leg	362 (97.6)
	Elsewhere	9 (2.4)
Does the person have hydrocele?	Yes	4 (2.1)
	No	187 (97.9)
Did the person experience any acute attack in the past 6 months?	Yes	266 (71.5)
	No	106 (28.5)
Is the person a known leprosy patient now or in the past?	Yes	7 (1.9)
	No	363 (98.1)
Did the person live for more than 10 years in areas at least 1000 m altitude?	Yes	349 (95.9)
	No	15 (4.1)
Does the person have signs and symptoms of onchocerciasis?	Yes	5 (1.3)
	No	367 (98.6)
Is the person known to have diabetes mellitus, heart or kidney disease?	Yes	19 (5.2)
	No	348 (94.8)
Has the person had surgery below the hip?	Yes	2 (0.5)
	No	368 (99.4)
Is there swelling of the face or breast or other part of the body than the leg?	Yes	9 (2.4)
	No	362 (97.6)
Does the person have knob, bump or lump on the lower leg?	Yes	38 (10.3)
	No	330 (89.7)
Does the person have moss on the lower leg?	Yes	202 (54.3)
	No	170 (45.7)
Does the person have joint fixation of the lower leg?	Yes	2 (0.5)
	No	370 (99.5)
Shoe wearing habits	Never	11 (3)
	Sometimes	304 (82.6)
	Always	53 (14.4)
Is there a family history of leg swelling?	Yes	215 (58.1)
	No	155 (41.9)
Wb123 test results at 30 min	Negative	372 (100)
Wb123 test results at 24 h	Negative	372 (100)
Expert diagnosis	Podoconiosis	344 (92.5)
	Other lymphoedema	28 (7.5)

Table 3 shows the results of univariate regression analysis. Of the 16 variables included, only 6 variables were found to be significant. Swelling that started from the lower leg, swelling of ≥1 y duration, experiencing an acute attack in the past 6 months and family history of lower leg swelling increased the odds of being a podoconiosis patient. However, a history of leprosy and swelling of other parts of the body were associated with a lower risk of being a podoconiosis patient. In addition, the presence of moss and the presence of a knob, bump or lump on the lower leg were significantly associated with podoconiosis in a χ^2^ test.

**Table 3.** Univariate logistic regression analysis

	Lymphedema cause			
Variable	Podoconiosis	Other	Unadjusted OR (95% CI)	p-Value
Sex (n=372)				
Male	180 (52.3)	11 (39.3)	1.7 (0.8 to 3.7)	0.19
Female	164 (47.7)	17 (60.7)	1.0	
Type of swelling (n=372)				
Unilateral	44 (12.8)	7 (25.0)	1.0	
Bilateral	300 (87.2)	21 (75.0)	0.4 (0.2 to 1.1)	0.08
Dose the person have swelling in the groin area (n=321)?			1.0	
Yes	84 (28.0)	2 (9.5)	3.7 (0.84 to 16.21)	0.08
No	216 (72.0)	19 (90.5)	1.0	
Does the swelling differ in size between the two legs?^a^				
Yes	93 (31.1)	10 (47.6)		0.095^b^
No	206 (68.9)	11 (52.4)		
Is the swelling limited to below the knee? (n=366)				
Yes	335 (98.8)	27 (100)	0.5 (0.2 to 1.2)	0.12
No	4 (1.2)	0 (0.0)	1.0	
Where did the swelling start from? (n=371)				
Lower leg	339 (98.8)	23 (82.1)	1.0	
Elsewhere	4 (1.2)	5 (17.9)	0.05 (0.01 to 0.22)	<0.001
Did the person experience an acute attack in the past 6 months? (n=372)				
Yes	253 (73.5)	13 (46.4)	3.2 (1.4 to 7.0)	0.003
No	91 (26.5)	15 (53.6)	1.0	
Is the person experiencing an acute attack during the interview? (n=370)				
Yes	5 (1.5)	1 (3.6)	0.4 (0.04 to 3.55)	0.411
No	337 (98.5)	27 (96.4)	1.0	
Is the person a known leprosy patient now or in the past? (n=370)				
Yes	3 (0.9)	4 (14.3)	0.05 (0.01 to 0.25)	<0.001
No	339 (99.1)	24 (85.7)	1.0	
Has the person lived for >10 y at areas >1000 m altitude? (n=364)				
Yes	323 (96.1)	26 (92.9)	1.91 (0.41 to 8.93)	0.41
No	13 (3.9)	2 (7.1)	1.0	
Does the person have signs or symptoms of onchocerciasis? (n=372)^a^				0.675^b^
Yes	5 (1.5)	0 (0.0)		
No	339 (98.5)	28 (100)		
Is the person known to have diabetes mellitus, heart or kidney disease? (n=367)				
Yes	17 (5.0)	2 (7.1)	0.67 (0.15 to 3.13)	0.627
No	322 (95.0)	26 (92.9)	1.0	
Is there a history of surgery below the hip? (n=370)^a^				0.854^b^
Yes	2 (0.6)	0 (0.0)		
No	340 (99.4)	28 (100)		
Is there swelling on any other part of the body? (n=371)				
Yes	6 (1.7)	3 (10.7)	0.15 (0.04 to 0.63)	0.01
No	337 (98.3)	25 (89.3)	1.0	
Does the person have a knob, bump or lump on the lower leg? (n=368)^£^				0.047^b^
Yes	38 (11.1)	0 (0.0)		
No	303 (88.9)	27(100)		
Does the person have moss on the lower leg? (n=372)			χ^2^=33.65	<0.001^c^
Yes	202 (58.7)	0 (0.0)		
No	142 (41.3)	28 (100)		
Does the person have joint fixation of the lower leg? (n=372)				0.885^b^
Yes	2 (0.6)	0 (0.0)		
No	342 (99.4)	28(100)		
Shoe wearing habits (n=368)				
Never	10 (2.9)	1 (3.7)	0.82 (0.08 to 8.10)	0.862
Sometimes	282 (82.7)	22 (81.5)	1.05 (0.35 to 3.17)	0.936
Always	49 (14.4)	4 (14.8)	1.0	
Family history of leg swelling?				
Yes	210 (61.4)	5 (17.9)	7.32 (2.72 to 19.72)	<0.001
No	132 (38.6)	23 (82.1)	1.0	
Duration of swelling				
>1 y	343 (99.7)	23 (85.2)	59.6 (6.4 to 555.6)	<0.001
≤1 y	1 (0.3)	4 (14.8)		

^a^Because of zero values we were unable to fit a logistic regression model.

^b^Fisher's exact test.

^c^χ^2^ with continuity correction.

The performance of the individual signs and symptoms is described in Table 4. In multivariate logistic regression, the best predictive model contained five variables (Table 5). People with lymphoedema and no history of leprosy had higher odds of podoconiosis (OR 19.42 [95% confidence interval {CI} 0.87 to 434.74]) than those with such a history. People with swelling in the groin area had lower odds of podoconiosis compared with those without (OR 0.11 [95% CI 0.02 to 0.64]). People without known chronic illness such as diabetes mellitus or heart or kidney disease had higher odds of podoconiosis compared with those with such diseases (OR 17.96 [95% CI 1.58 to 204.02]). People without a family history of leg swelling had lower odds of podoconiosis than those with a family history (OR 0.038 [95% CI 0.01 to 0.22]).

**Table 4.** Performance of individual signs and symptoms

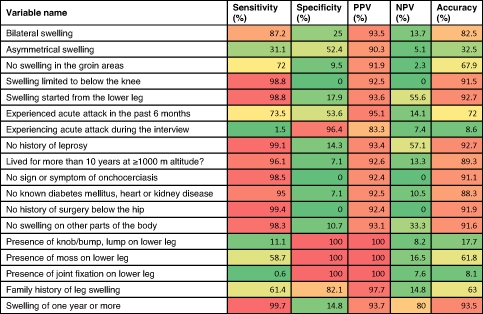

**Table 5.** Point estimates and 95% CIs for the predictors from the model having the highest sensitivity and specificity

Variables	Adjusted OR (95% CI)
Does the person have moss on the lower leg? (n=372)^a^	
Yes	
No	
Is the person a known leprosy patient now or in the past? (n=370)	
Yes	1.0
No	19.42 (0.87 to 434.74)
Does the person have swelling in the groin area? (n=321)	
Yes	0.11 (0.02 to 0.64)
No	1.0
Is the person known to have diabetes mellitus, heart or kidney disease? (n=367)	
Yes	1.0
No	17.96 (1.58 to 204.02)
Family history of leg swelling? (n=370)	
Yes	1.0
No	0.038 (0.01 to 0.22)

^a^This model also includes moss, whose OR cannot be reliably estimated due to the absence of patients for which with no moss and also tested negative for podoconiosis.

**Figure 2.**
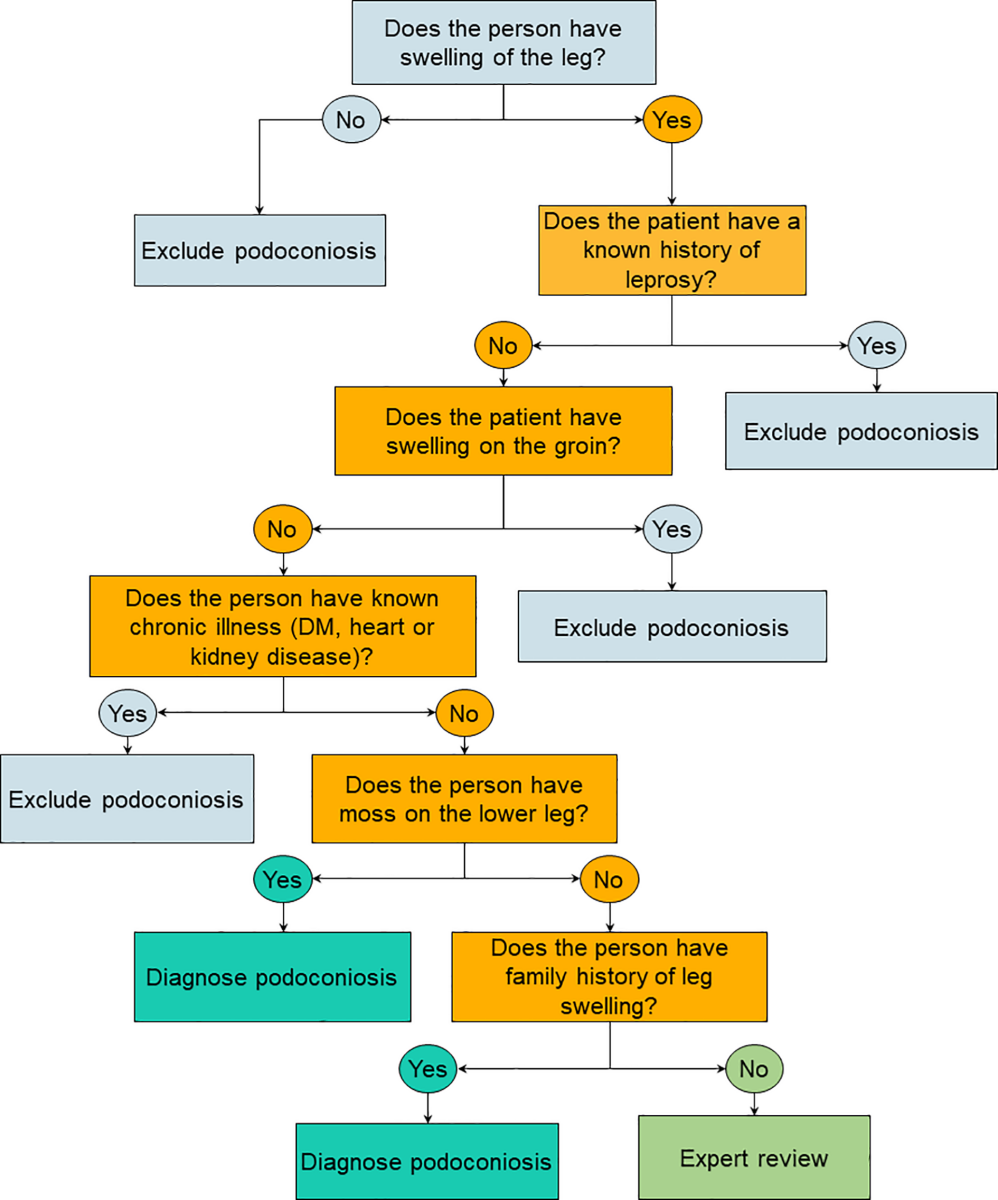
Algorithm for the diagnosis of podoconiosis in persons with lower leg lymphoedema.

As depicted in Figure 2, the best performing set of symptoms and signs was the presence of moss on the lower legs and a family history of leg swelling, plus the absence of current or previous leprosy, plus the absence of swelling in the groin, plus the absence of chronic illness (such as diabetes mellitus or heart or kidney diseases) with the order indicated in Figure 2. The overall sensitivity of the algorithm was 91% (95% CI 87.6 to 94.4) and specificity was 95% (95% CI 85.45 to 100). The positive predictive value and negative predictive value were 99.6% (95% CI 98.8 to 100) and 41.2% (95% CI 28.54 to 57.82), respectively.

## Discussion

The clinical, social and economic burden caused by podoconiosis is enormous in endemic countries.^4,24–27^ Recognition of this has led us to develop a simple clinical algorithm to help primary healthcare workers accurately diagnose podoconiosis. We found that the presence of moss on the lower legs and a family history of leg swelling, the absence of current or previous leprosy, the absence of swelling in the groin and the absence of chorionic illness (such as diabetes mellitus or heart or kidney diseases) can identify people with lymphoedema with a high probability of podoconiosis. The algorithm has a favourable performance in identifying podoconiosis cases across settings of varying endemicity. We believe this algorithm and the screening questions are acceptable for practitioners in endemic countries because they are signs and symptoms classically associated with podoconiosis and confirmed by previous studies.^25,28^

Misdiagnosis and delayed diagnosis of podoconiosis continue to be a challenge in endemic countries. This means that patients with podoconiosis are denied the treatment and care they deserve, contributing to significant morbidity, reduced quality of life and reduced productivity.^29^ In the absence of a rapid, reliable and effective point-of-care diagnosis, the need for a simple clinical algorithm to screen people with lymphoedema that differentiates people with and without podoconiosis is important.^7^ The algorithm presented here is an efficient method for diagnosing podoconiosis, comprising five simple questions easily assessed by primary healthcare workers. The algorithm identifies patients with lymphoedema of the lower limbs in whom a diagnosis of podoconiosis is likely and who should therefore be managed as such. This will allow simple interventions to be implemented in the primary healthcare setting and has the potential to significantly reduce morbidity and improve quality of life. As many patients with podoconiosis live in remote areas and may be unable to travel to a secondary healthcare centre, the algorithm may allow them to be diagnosed and managed in their local community.^25^ For those patients in whom a diagnosis is deemed unlikely or inconclusive according to the algorithm, the healthcare worker might refer them to the next level of the healthcare system to perform further detailed assessment and appropriate investigation.

The algorithm, which can be used by healthcare workers with minimal training, correctly identified 91% of people with podoconiosis and 95% of those without podoconiosis. Of the total 372 participants included in our study, the majority (344 [92.5%]) were podoconiosis cases and only 28 people with lymphoedema due to other causes were identified. Although we included one LF-endemic district, none of the people with lymphoedema had a positive Wb123 test. Nonetheless, it is worth mentioning that previous LF-specific nationwide studies and subsequent geostatistical models have shown that LF is focal and its transmission intensity is low in Ethiopia.^9,30,31^ We thus expect that the inclusion of an LF-specific antigen-based diagnostic test into the clinical algorithm in areas highly endemic for LF may increase its performance by increasing its specificity. Therefore it is likely that any particular algorithm will be site specific and it will probably be necessary to improve the performance by including different differential diagnoses for different geographical areas. For example, one of the questions concerns a history of leprosy, which would only be valid in areas where leprosy is endemic. However, we believe that the principles underlying this approach to the diagnosis of podoconiosis could be applied more widely. Further studies in areas with different epidemiological patterns and a variable presence of confounding diseases are needed to see if this is the case.

Our study has several strengths, especially the simplicity of the signs and symptoms included in our clinical algorithm, which allow it to be implemented by primary healthcare workers with minimal training. Also, we included districts with a range of epidemiological profiles of podoconiosis and other potential causes of lymphoedema. Nevertheless, there are some limitations to our work. First, we included signs and symptoms that are closely associated with podoconiosis and major tropical lymphoedemas. However, there are several other causes of lymphoedema associated with different clinical features.^32^ It is possible that the addition of one or more pertinent additional question that was not included in our study could increase the performance of the algorithm. Second, we used clinical diagnosis by healthcare workers as the gold standard for diagnosis of podoconiosis. Podoconiosis is a clinical diagnosis and there is no point-of-care diagnostic test. However, to support clinical decision-making by experienced healthcare providers we performed Wb123 tests to potentially exclude LF. Limitations related to the Wb123 test should also be put into context. First, the Wb123 test cannot distinguish current active infection from past infection in individuals from endemic areas.^15^ This limitation may not be of particular importance in our case since identification of individuals with current or previous disease would help in excluding cases due to LF. Second, the Wb123 test has a lower specificity and sensitivity compared with the FTS. Although the studied areas are generally low LF prevalence areas, we might have missed LF-positive cases with lymphoedema. Therefore, for future studies we suggest the use of FTS or the use of both tests based on the epidemiology of other confounding diseases.^15,33^

The majority of individuals included in the study had a diagnosis of podoconiosis and only a small number of patients had other types of lymphoedema. In view of this, we were unable to separate the data into training and test groups, as this would have left too few patients with non-podoconiosis-related lymphoedema in each group. As a result, we have not yet been able to fully validate the algorithm. We are planning to do this in future work and include clinical data from other endemic regions to further assess its accuracy. Ideally the algorithm proposed here should be prospectively evaluated in multiple countries and geographic regions, as our study was conducted in just one region of Ethiopia.

## Conclusions

Early diagnosis and timely morbidity management of podoconiosis require the development of a rapid, accurate, point-of-care diagnostic test. In the absence of such a test, a standardized algorithm using signs and symptoms, such as the one we propose here, will help in early diagnosis and treatment of podoconiosis, reducing the suffering of people with podoconiosis and improving their quality of life.
